# Employing the Gini coefficient to measure participation inequality in treatment-focused Digital Health Social Networks

**DOI:** 10.1007/s13721-016-0140-7

**Published:** 2016-10-27

**Authors:** Trevor van Mierlo, Douglas Hyatt, Andrew T. Ching

**Affiliations:** 1 Henley Business School, University of Reading, Greenlands, Henley-on-Thames, RG9 3AU UK; 2 Evolution Health Systems Inc., 900 King Streeet West, Suite 401, Toronto, M5V 3H5 Canada; 3 Rotman School of Management, University of Toronto, 105 St George Street, Toronto, M5S 3E6 Canada

**Keywords:** Online support groups, Social networks, Gini coefficient, Econometrics, Superusers

## Abstract

Digital Health Social Networks (DHSNs) are common; however, there are few metrics that can be used to identify participation inequality. The objective of this study was to investigate whether the Gini coefficient, an economic measure of statistical dispersion traditionally used to measure income inequality, could be employed to measure DHSN inequality. Quarterly Gini coefficients were derived from four long-standing DHSNs. The combined data set included 625,736 posts that were generated from 15,181 actors over 18,671 days. The range of actors (8–2323), posts (29–28,684), and Gini coefficients (0.15–0.37) varied. Pearson correlations indicated statistically significant associations between number of actors and number of posts (0.527–0.835, *p* < .001), and Gini coefficients and number of posts (0.342–0.725, *p* < .001). However, the association between Gini coefficient and number of actors was only statistically significant for the addiction networks (0.619 and 0.276, *p* < .036). Linear regression models had positive but mixed *R* ^2^ results (0.333–0.527). In all four regression models, the association between Gini coefficient and posts was statistically significant (*t* = 3.346–7.381, *p* < .002). However, unlike the Pearson correlations, the association between Gini coefficient and number of actors was only statistically significant in the two mental health networks (*t* = −4.305 and −5.934, *p* < .000). The Gini coefficient is helpful in measuring shifts in DHSN inequality. However, as a standalone metric, the Gini coefficient does not indicate optimal numbers or ratios of actors to posts, or effective network engagement. Further, mixed-methods research investigating quantitative performance metrics is required.

## Introduction

Technology is now an important component of the healthcare ecosystem, and both computer and management science are playing a greater role in analyzing data to measure efficiencies (Chaudhry et al. 2006; Harrison et al. 2007; Holden and Karsh 2010). The digital health industry is maturing, and as a result, highly tailored evidence-based behavior change programs are increasingly available to consumers through pharmaceutical companies, non-profit organizations, insurers, private corporations, and government entities.

A component of these programs is Digital Health Social Networks (DHSNs), otherwise known as bulletin boards or peer-to-peer support groups. While there are still no firm conclusions on how to determine their efficaciousness (Eysenbach et al. 2004; Graham et al. 2015), the general consensus is that social support and knowledge sharing increases patient education, enhances self-management, and decreases burden on existing health services (Bender et al. 2013; Brennan et al. 1995; Cobb et al. 2011; Conrad et al. 2016; Ploderer et al. 2013; Takahashi et al. 2009; Wicks et al. 2012; Wright 2002).

Hypothetically, an ideal DHSN would consist of members who are equally engaged. In reality, network participation is unequal. An issue is that other than observing a network’s number of actors and number of posts, there are few metrics that can be used to identify participation inequality.

To address this issue, some research has sought to define actor roles (Carron-Arthur et al. 2015; Cleary and Stanton 2015; Cunningham et al. 2008; Jones et al. 2011; Selby et al. 2010; van Mierlo et al. 2012). By systematically categorizing participants, taxonomies can give insight into how various actors in complex networks function in relation to one another.

Other research has explored network topologies. Some of these studies have employed traditional social network analysis and method that focuses on nodes, ties, density of relationships, and degree centrality (Cobb et al. 2010; Urbanoski et al. 2016). Other streams have examined marketing rules of thumb (van Mierlo 2014), latent semantic analysis (Myneni et al. 2013), natural language processing (Wang et al. 2015), or the phenomenon of power laws (Carron-Arthur et al. 2014; van Mierlo et al. 2015).

As DHSNs shift into mainstream healthcare delivery it will be important to develop metrics that help managers assess growth, sustainability, and participation equality (Healey et al. 2014; Stearns et al. 2014). While the quality of DHSN content is important and is rooted in behavioral science, quantitative methods to analyze the health of a network may come from established theoretical constructs in economics and computer science.

Through measuring 222 quarters of participation from 15,181 actors from four separate DHSNs, this paper investigates whether the Gini coefficient, an economic measure of statistical dispersion traditionally used to measure income distribution in populations, can be employed as a management tool to help measure inequality of member participation over time.

### The Lorenz curve

**Fig. 1:**
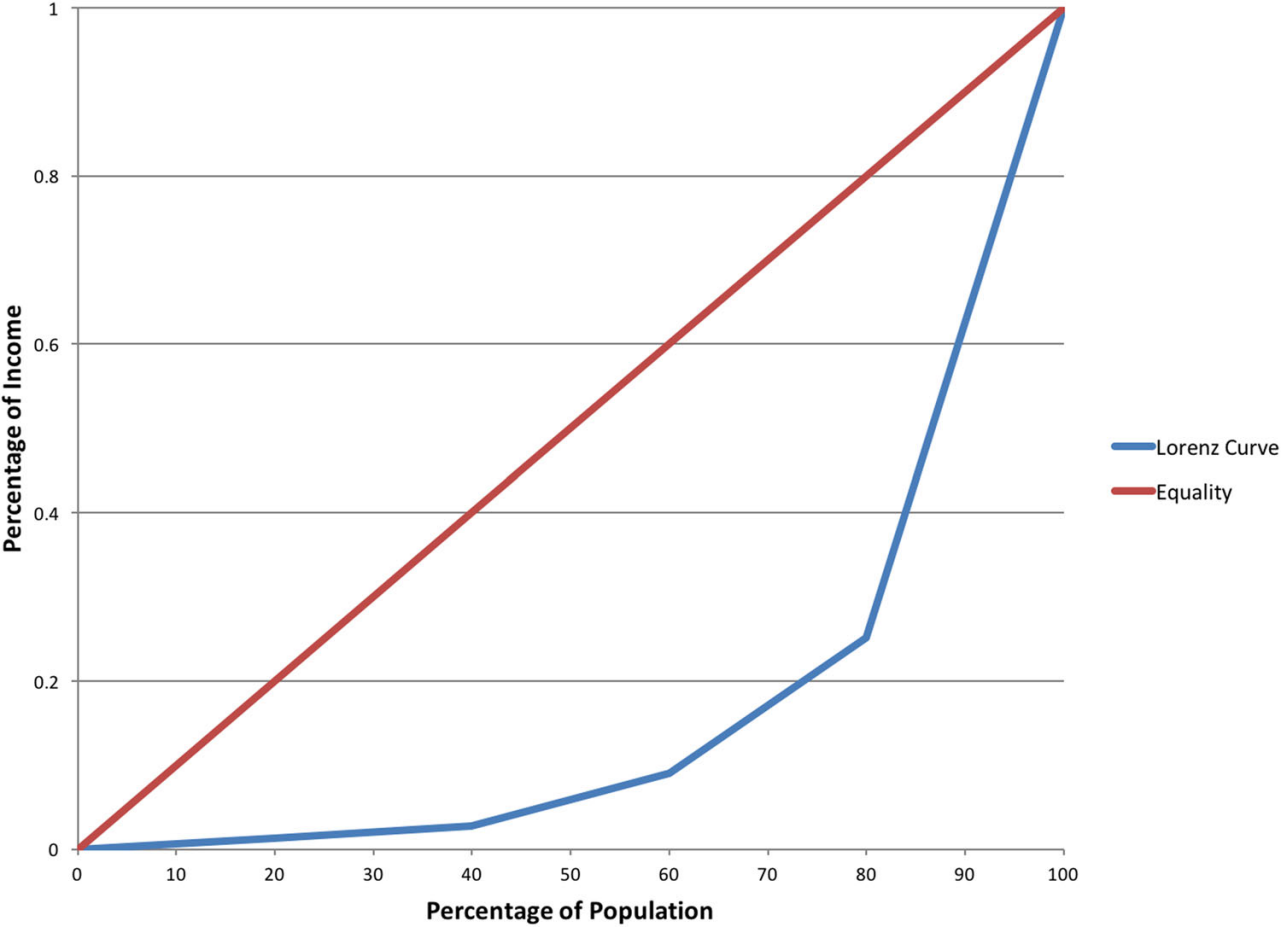
A Lorenz curve

In economics, the Lorenz curve is a popular method that illustrates income distribution (Lorenz 1905). Graphically, the *y*-axis represents the percentage of income in an economy, and the *x*-axis represents cumulative income distribution in the total percentage of households (Fig. 1).

In a perfectly equal economy, all citizens share the same income, and the Lorenz curve would resemble the red line in Fig. 1, where *y* = *x*. Most economies are not equal. In Fig. 1, the Lorenz curve (blue line) illustrates economic inequality. Here, approximately 20 % of households receive 1 % of income, 40 % receive 3 %, 60 % receive 8 %, and 80 % receive 25 %.

### The Gini coefficient

Developed by Corrado Gini in 1912 (Gini 1912), the Gini coefficient is an inequality measure based on the Lorenz curve. Specifically, it measures the distance between the Lorenz curve and perfect equality (Bellu and Liberati 2006). A Gini coefficient of 1 represents an economy where a single individual generates all income, whereas a Gini coefficient of 0 represents an economy where all citizens share the same income.

If translated to DHSNs, a Gini coefficient of 1 would represent a network where one individual created all posts. Alternatively, a Gini coefficient of 0 would represent a social network where all members authored the same number of posts.

To our knowledge, Gini coefficient numerical and visual outputs have yet to be applied to assess participation inequality in DHSNs. However, the method has been utilized in other studies.

For example, a 2005 study accessed U.S. census data to measure Personal Computer (PC) ownership inequalities amongst whites and African Americans at national, regional, and state levels (Chakraborty and Bosman 2005). Results indicated that although decreasing overall, PC ownership inequality is substantially smaller among white households. A strength of the study was the use of the Lorenz curve and Gini coefficient to graphically illustrate variations in income and PC ownership within and between the two groups.

Gini coefficient numerical scores and their visual representations were also utilized in a 2002 Statistics Canada research study depicting the digital divide, or cumulative internet usage amongst differing household income deciles (Sciadas 2002). Results indicate that as time progresses, the Gini coefficient is decreasing and the digital divide is closing. However, graphical outputs show that the shift is mainly attributable to middle-income groups, while lower-income and upper-income groups remain fairly stable.

Although there are other uses in research, a final example is a 2010 study analyzing university rankings. This study employed the Gini coefficient to assess whether academic institutions were becoming increasingly unequal (Halffman and Leydesdorff 2010). Ranking data mainly consisted of weighted contributions of total number of publications and citations, and findings indicate that contrary to popular belief, the 500 universities that publish at greatest frequency were becoming more equal in terms of output.

## Methods

Data were extracted from the DHSNs of four interventions: AlcoholHelpCenter.net (AHC), DepressionCenter.net (DC), PanicCenter.net (PC), and StopSmokingCenter.net (SSC). All four digital health programs are based on state-of-the-art best practice guidelines, and have been extensively evaluated in the literature (Cunningham 2009, 2012; Cunningham et al. 2006a, b; Cunningham and van Mierlo 2009; Cunningham et al. 2009, 2010; Davis 2007; Doumas et al. 2009; Farvolden et al. 2003, 2005, 2009; McDonnell et al. 2011; Rabius et al. 2008). Table 1 outlines study duration, number of participants, and other descriptive data.

**Table 1:** Intervention and DHSN characteristics

Intervention	Social network launch date	Data acquisition date	Number of days active	Number of quarters	Number of subjects registered in program	Number of actors *N* (%)	Number of actor posts^b^
AHC	Dec 26, 2005	Dec 31, 2015	3658	41^a^	5049	1085 (21.5 %)	21,202
DC	Feb 6, 2003	Dec 31, 2015	4712	52	11,675	2074 (17.8 %)	20,513
PC	Jan 23, 2002	Dec 31, 2015	5091	56	9783	3593 (36.7 %)	61,861
SSC	Sep 26, 2001	Dec 31, 2015	5210	58	52,396	8451 (16.1 %)	522,160
Total	n/a	n/a	18,671	222	78,903	15,181 (19.2 %)	625,736
Mean	n/a	n/a	4688	55.5	19,726	3795 (19.2 %)	156,434

^a^40 quarters used in the analysis

^b^Moderator posts removed

For the study duration, a staff of trained moderators monitored and maintained the four networks. Key moderator roles included ensuring compliance with network policies and user agreements, answering actor questions, guiding discussions when deemed appropriate, and offering technical assistance. For the purpose of analysis, moderator posts were removed from the dataset.

For each network, data sets were divided into annual quarters. Within each quarter, the total number of posts by each unique actor was calculated, and actors were ranked according to level of contribution. Next, the population in each quarter was divided into quintiles. Finally, Gini coefficients for each quarter were calculated. Pearson correlations and linear regression were used to assess the strength of relationships between Gini coefficient, actors, and posts.

All data collection policies and procedures adhered to international privacy guidelines (European Parliament and of the Council on the Protection of Individuals with Regard to the Processing of Personal Data and on the Free Movement of Such Data 2002; Office of the Privacy Commissioner of Canada 2000; US Department of Health and Human Services 2003) and were in accordance with the Helsinki Declaration of 1975, as revised in 2008 (World Medical Association 2008). The study was consistent with University Research Ethics Committee procedures at Henley Business School, University of Reading, and was exempt from full review.

## Results

**Fig. 2:**
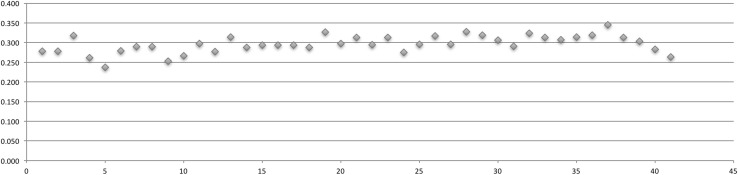
AHC Gini coefficient over 41 quarters

**Fig. 3:**
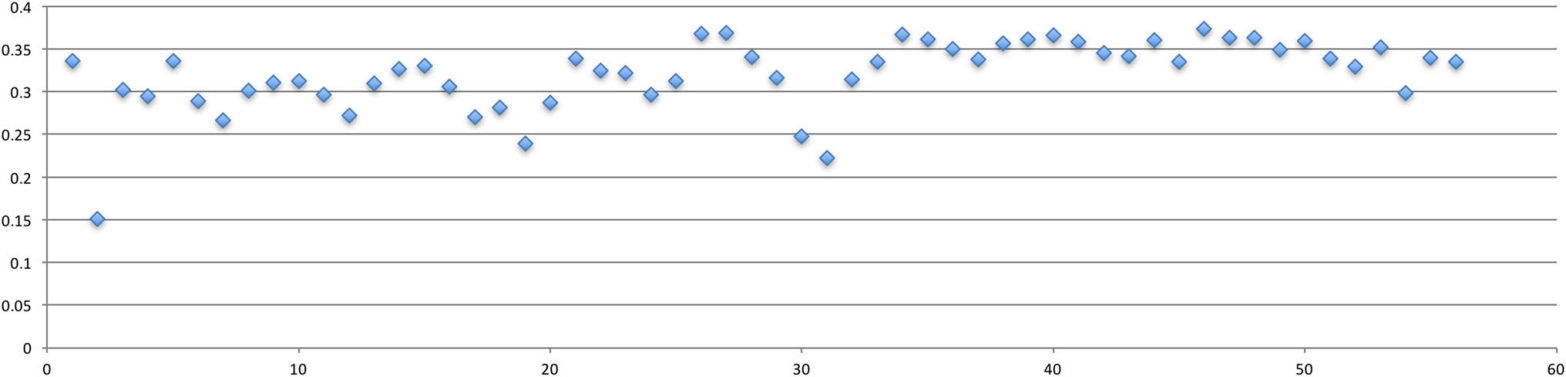
PC Gini coefficient over 56 quarters

**Fig. 4:**
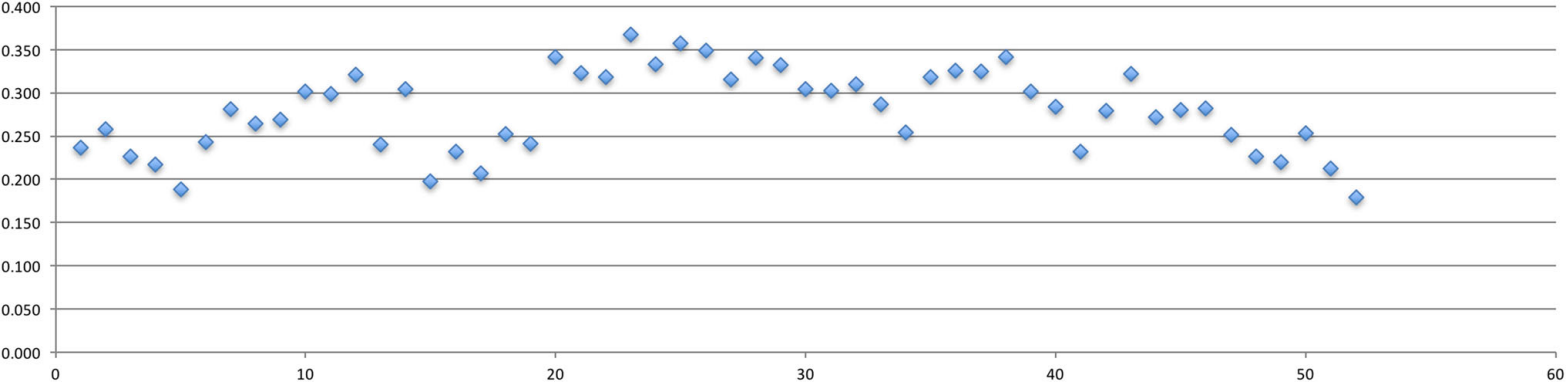
DC Gini Coefficient over 52 quarters

**Fig. 5:**
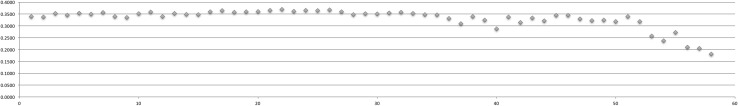
SSC Gini coefficient over 52 quarters

Table 2 displays the relationships between each quarter’s Gini coefficient, number of actors, and number of posts (Table 2). For each network, fluxions in quarterly shifts in the Gini coefficient were graphed (Figs. 2, 3, 4, 5).

**Table 2:** Actors, posts, Gini coefficient

	AHC			DC			PC			SSC		
	Actors	Posts	Gini	Actors	Posts	Gini	Actors	Posts	Gini	Actors	Posts	Gini
01-Q3										44	1361	0.340
01-Q4										43	1330	0.338
02-Q1							17	383	0.336	202	9665	0.353
02-Q2							19	41	0.151	310	11,373	0.346
02-Q3							52	581	0.302	376	15,336	0.354
02-Q4							91	530	0.295	504	16,903	0.349
03-Q1				18	66	0.236	152	590	0.336	441	16,752	0.356
03-Q2				44	152	0.258	133	835	0.289	341	10,645	0.339
03-Q3				61	184	0.226	201	1220	0.266	327	8772	0.336
03-Q4				89	263	0.217	178	1610	0.301	295	11,044	0.351
04-Q1				94	264	0.189	226	2434	0.310	320	13,787	0.358
04-Q2				76	225	0.243	227	2131	0.313	289	8250	0.339
04-Q3				70	460	0.281	282	2092	0.296	331	9115	0.352
04-Q4				124	615	0.264	221	1451	0.273	412	9626	0.348
05-Q1				209	1016	0.269	361	3125	0.310	722	20,914	0.348
05-Q2				196	1428	0.302	289	3750	0.327	666	24,135	0.359
05-Q3				45	397	0.299	195	2333	0.331	661	22,907	0.365
05-Q4				66	655	0.321	83	845	0.306	536	19,125	0.357
06-Q1	18	128	0.288	88	311	0.241	95	536	0.270	816	24,776	0.359
06-Q2	27	257	0.318	72	594	0.304	68	446	0.282	696	28,684	0.360
06-Q3	14	88	0.280	47	104	0.198	87	337	0.239	579	28,316	0.365
06-Q4	10	37	0.254	48	165	0.232	89	479	0.287	578	26,147	0.369
07-Q1	21	95	0.259	53	142	0.207	95	1226	0.339	771	27,947	0.361
07-Q2	21	169	0.290	72	269	0.253	97	1042	0.324	571	23,763	0.365
07-Q3	22	82	0.266	63	264	0.241	56	480	0.322	381	21,878	0.364
07-Q4	23	209	0.264	78	1214	0.341	71	484	0.296	359	14,518	0.368
08-Q1	19	69	0.223	64	775	0.323	62	431	0.312	366	17,296	0.360
08-Q2	14	29	0.193	58	388	0.318	59	1370	0.368	307	9729	0.347
08-Q3	24	100	0.266	43	1268	0.368	57	2201	0.369	241	8674	0.352
08-Q4	30	207	0.220	57	1638	0.333	71	1216	0.340	196	6455	0.351
09-Q1	39	301	0.295	52	865	0.357	81	750	0.316	250	7905	0.354
09-Q2	27	209	0.284	35	521	0.349	62	266	0.247	195	7145	0.358
09-Q3	40	213	0.264	45	387	0.316	62	265	0.222	188	5356	0.352
09-Q4	34	258	0.260	44	1244	0.340	71	709	0.314	151	4540	0.348
10-Q1	27	176	0.263	41	715	0.332	81	1228	0.335	157	4298	0.346
10-Q2	29	321	0.326	42	543	0.305	54	1843	0.367	119	2867	0.331
10-Q3	30	242	0.283	34	347	0.303	62	1930	0.361	118	2323	0.309
10-Q4	48	779	0.316	39	281	0.310	67	1420	0.350	118	1944	0.339
11-Q1	42	445	0.289	48	303	0.286	71	1907	0.338	109	1529	0.324
11-Q2	30	291	0.315	44	224	0.254	64	1900	0.356	91	838	0.287
11-Q3	30	237	0.261	23	253	0.319	50	1527	0.361	79	1176	0.337
11-Q4	53	340	0.279	31	227	0.326	55	2063	0.366	94	1074	0.315
12-Q1	56	563	0.318	27	280	0.325	51	1507	0.358	100	1365	0.334
12-Q2	55	532	0.286	20	225	0.341	42	704	0.345	80	1106	0.322
12-Q3	52	1330	0.326	8	77	0.301	49	422	0.342	70	1294	0.345
12-Q4	58	787	0.313	21	107	0.284	52	1318	0.360	64	1076	0.345
13-Q1	57	707	0.304	20	62	0.232	41	778	0.335	72	890	0.329
13-Q2	59	447	0.289	16	78	0.279	30	1121	0.373	77	1261	0.322
13-Q3	61	888	0.330	25	161	0.322	28	985	0.363	57	1058	0.324
13-Q4	88	983	0.312	25	98	0.271	48	1253	0.363	72	765	0.318
14-Q1	84	746	0.305	36	173	0.280	29	611	0.349	90	1181	0.339
14-Q2	65	722	0.314	26	129	0.282	41	711	0.359	54	675	0.318
14-Q3	62	767	0.318	18	58	0.252	28	367	0.339	60	261	0.257
14-Q4	60	868	0.345	27	97	0.227	32	419	0.329	75	262	0.237
15-Q1	69	562	0.313	18	41	0.220	29	443	0.352	78	335	0.272
15-Q2	50	395	0.303	20	67	0.254	23	148	0.299	55	156	0.210
15-Q3	51	409	0.283	21	64	0.213	44	663	0.340	64	151	0.205
15-Q4	33	238	0.263	15	29	0.179	50	404	0.335	49	106	0.181

Summary statistics were computed to identify means and standard deviation for each network’s number of actors, number of posts, and Gini coefficient (see Table 3).

**Table 3:** Summary statistics

	AHC	DC	PC	SSC
Actors				
Minimum	10	8	17	43
Maximum	88	209	361	2323
Range	78	201	344	2280
*μ*	40.8	51.0	89.8	304.7
SD	19.6	38.9	75.16	347.3
Posts				
Minimum	29	29	41	106
Maximum	1330	1638	3750	28,684
Range	1301	1609	3709	28,578
*μ*	405.7	369.5	1104.7	9002.8
SD	308.6	391.4	780.9	9049.5
Gini coefficient				
Minimum	0.19	0.18	0.15	0.18
Maximum	0.35	0.37	0.37	0.37
Range	0.15	0.19	0.22	0.19
*μ*	0.287	0.279	0.321	0.332
SD	0.032	0.058	0.042	0.041

The DHSN with the least number of actors in any single quarter was the DC (*n* = 8), and the SSC was the DHSN with the greatest number of actors in any given quarter (*n* = 2323). Mean number of actors varied (*n* = 40.8–304.7), as did standard deviation (*n* = 19.6–347.3).

The AHC and DC had the least number of posts in any single quarter (*n* = 29), and the SSC was the DHSN with the greatest number of posts in any given quarter (*n* = 28,684). Mean number of posts varied (*n* = 405.7–9002.8), as did standard deviation (*n* = 308.6–9049.5).

In regards to Gini coefficient, three of the four DHSNs had at least one quarter with the highest level of inequality (0.37). Interestingly, each of the four social networks had quarters with similar lowest levels of inequality (0.15–0.19). Range of Gini coefficient varied slightly (0.15–0.22), however, mean (0.287 and 0.332) and standard deviation (0.032–0.058) did not.

Pearson correlations were then calculated to compare number of actors, posts, and Gini coefficient (see Table 4).

**Table 4:** Pearson correlations between Gini coefficient, actors, and posts

	Actors (sig)	Posts (sig)
AHC		
Actors	1	0.835 (0.01)
Gini	0.619 (0.001)	0.725 (0.01)
DC		
Actors	1	0.551 (0.01)
Gini	−0.028 (0.842)	0.590 (0.01)
PC		
Actors	1	0.676 (0.01)
Gini	−0.206 (0.127)	0.342 (0.01)
SSC		
Actors	1	0.527 (0.00)
Gini	0.276 (0.036)	0.575 (0.00)

Pearson correlations computed positive and statistically significant relationships between number of actors and number of posts (0.527–0.835, *p* < .001), and Gini coefficient and number of posts (0.342–0.725, *p* < .001). However, the relationship between Gini coefficient and number of actors was only positive and statistically significant for the addiction networks (0.619 and 0.276, *p* < .036).

Multiple regressions were then computed to examine the association between the Gini coefficient (dependent variable), and independent variables actors and posts (see Table 5).

**Table 5:** Linear regression

	AHC	DC	PC	SSC
Constant				
*β*	0.255	0.269	0.309	0.309
*t*-stat	28.793	34.267	41.888	47.138
sig	0.000	0.000	0.000	0.000
Actors				
*β*	7.503E−005	−0.001	0.000	0.000
*t*-stat	0.222	−4.305	−5.934	−0.291
sig	0.825	0.000	0.000	0.772
Posts				
*β*	7.164E−005	0.000	4.758E−005	2.680E−006
*t*-stat	3.346	7.381	6.528	4.600
sig	0.002	0.000	0.000	0.000
*R* ^2^	0.527	0.527	0.469	0.333
Collinearity statistics (tolerance)	0.303	0.697	0.543	0.723
Collinearity statistics (VIF)	3.297	1.435	1.843	1.384
Durbin–Watson	1.546	1.092	1.638	0.312

Linear regression models had mixed *R* ^2^ results (0.333–0.527). In all four regression models, the association between Gini coefficient and posts were statistically significant (*t* = 3.346–7.381, *p* < .002). However, as opposed to Pearson correlations, the relationship between Gini coefficient and number of actors was only statistically significant in the two mental health networks (*t* = −4.305 and −5.934, *p* < .000).

In regards to collinearly, tolerance was all above 0.10 (0.303–0.723), and variance inflation factors were moderately correlated (1.384–3.297), indicating that multicollinearity is not a concern. However, as they did not approach a score of 2.0, Durbin–Watson statistics indicated the presence of autocorrelation (0.312–1.638).

## Discussion

The results of this study generate several unique contributions to DHSN research, all of which require further investigation.

### Detecting shifts in social network inequality

From both visual and quantitative perspectives, the Gini coefficient was effective in identifying shifts and trends in inequality. However, as a standalone metric, shifts in the Gini coefficient can be deceptive as coefficient of 0.33 can be calculated from a network of 29 actors who created 321 posts (see Table 2, AHC period 10-Q2), or a network of 119 actors who created 2867 posts (see Table 2, SSC period 10-Q2).

Future research in developing metrics to determine social network inequality should incorporate the Gini coefficient, but also test ratios pertaining to number of posts per actor, number of posts per Gini score, number of actors to Gini score, or various combinations.

### Use of the Gini coefficient as a management tool

During the process of the study, informal qualitative in-person interviews were conducted with moderators and other management, and the visual depictions of shifts in quarterly Gini coefficient were deemed particularly helpful when recalling effects of technical outages, policy changes, the implementation of management issues and techniques, and dynamics of personalities within groups of actors.

In these informal meetings, discussion often centered on the status and intensity of participation from Superusers, actors who generate the greatest amount of network externalities (van Mierlo et al. 2012). Strategies to increase equality and engage non-Superusers were also discussed. Future research may investigate the value of utilizing the Gini coefficient as a tool to assist with network management.

### Insights into social network utility and function

While there were consistent and statistically significant associations between Gini coefficient and number of posts in the Pearson correlations and the regressions, the associations between Gini coefficient and number of actors was inconsistent. It is interesting to note that this inconsistency was consistent for the two addiction DHSNs (AHC and SSC), and the two mood disorder DHSNs (DC and PC), and may potentially lend insight in quantifying the utility and function of DHSNs that promote different therapeutic approaches.

For example, the AHC and SSC interventions focus on addictions, and the theoretical approach to treatment in these programs is the Stages of Change (Prochaska et al. 2008) and Structured Relapse Prevention (Sanchez-Craig 1995). Both treatment approaches are designed to assist users in developing coping skills to assist with strong, yet relatively short-term cravings. This is reflected in program content, which consists of short exercises that offer brief, yet tailored feedback on how to deal with addiction issues in specific situations. Prior research indicates that the content and tone within the interventions’ DHSN reflects this.

A 2010 mixed-methods study on the SSC social network (Selby et al. 2010) analyzed demographic and smoking characteristics for both actors and non-actors, and qualitative analyses were conducted to explore themes in message content. Results indicated that the most frequent first posts were from new actors who were struggling with their quit attempts, and 90.6 % of responses to these posts were from experienced actors. The authors concluded that social support in the network was particularly beneficial to many new actors who short-term, time-sensitive assistance.

Conversely, the two mental health interventions (DC and PC) are heavily focused on Cognitive Behavioral Therapy (CBT) (Herbert and Forman 2011). At the core of each intervention are nine sessions that are designed to take a minimum of 9 weeks to complete. Members are given homework between each session, which involves intensive experiments, journaling, and self-reflection.

A 2013 mixed-methods doctoral thesis on the DC social network (Sugimoto 2013) found that DC actors generally sought informational support, emotional support, coaching support, and social companionship over long periods of time. Actors providing emotional support created an average of 8.3 posts, and those actors seeking support created an average of 5 posts. The author concluded that actor participation and social support in the network was long-term, and contributed to the everyday lives of actors.

### Future directions

Future research may investigate potential relationships or patterns between Gini coefficient and number of actors in DHSNs leveraging differing therapeutic approaches. While this study focused on calculating the Gini coefficient over annual quarters, future research may experiment with calculating the Gini coefficient over shorter or longer time periods.

### Strengths and limitations

The main strength of this paper was the number of study participants, the extensive longevity of the DHSNs, the number of posts, and the four separate indications, and that half of the social networks in the study were focused on mental health, and the other half addictions. We are not aware of any other study in the healthcare literature with such an extensive and complete data set.

Both a strength and limitation is that the populations analyzed are self-selecting populations that actively sought help. In the context of this study, it was helpful to have data sets of active and engaged participants. However, these results will not be indicative of populations of patients in health plans, hospital networks, or mass public health campaigns.

## Conclusion

The Gini coefficient is helpful in measuring shifts in DHSN inequality. However, as a standalone metric, the Gini coefficient may be misleading as does not indicate optimal numbers or ratios of actors to posts, or effective network engagement.

From a management perspective, the Gini coefficient may be leveraged as a tool to assist moderators in detecting trends, or as a training tool to help explain how network inequality fluctuates.

The results to this study may support prior mixed-methods research on two of the four social networks (Selby et al. 2010; Sugimoto 2013), which found differences in social network utility, functionality, and tone.

Further research investigating quantitative scoring techniques, performance metrics, and optimization benchmarks is required.
